# Impact of soil surface properties on soil swelling of different soil layers in collapsing wall of Benggang

**DOI:** 10.1371/journal.pone.0280729

**Published:** 2023-02-02

**Authors:** Zhi Zhang, Lexing You, Jinshi Lin, Yunbo Wu, Honglin Zhong, Jie Chen, Fangshi Jiang, Yue Zhang, Ming-Kuang Wang, Yanhe Huang

**Affiliations:** 1 Jinshan Soil and Water Conservation Research Center, Fujian Agriculture and Forestry University, Fuzhou, Fujian, China; 2 Fujian Agriculture and Forestry University, Fuzhou, Fujian, China; Jinan University, CHINA

## Abstract

Benggang is one of the most serious soil erosion problems in tropical and subtropical areas in southern China. Little work has been reported on the surface properties of soil colloidal particle and its influence on soil swelling of different soil layers in collapsing wall of Benggang. In this present work, the effects of sodium concentration on soil swelling, and the correlations between soil swelling rates and soil colloidal surface properties were comprehensively evaluated by carefully examining soil physicochemical properties and soil colloidal surface properties of red, sandy and detritus soil layers from a collapsing wall. Our results showed that the soil swelling rates of red, sandy and detritus soil layers all exponentially decreased with increasing initial water contents. The relationship between soil swelling rate and the thickness of shear plane showed an extremely significant negative correlation for red soil layer and no correlation for sandy and detritus soil layers. Moreover, the elevating sodium concentrations reduced the thickness of shear plane from 39.69 to 0.76 nm for red soil layer, followed from 22.56 to 0.79 nm for sandy soil layer and from 18.61 to 0.64 nm for detritus soil layer. These findings indicated that the soil particle interactions played a crucial role in the development and occurrence of Benggang. This work will be helpful in understanding the mechanisms of soil mass loss on the gully head and collapsing wall of Benggang.

## Introduction

Benggang is a special type of soil erosion phenomenon and landform caused by the combined action of hydraulic and gravity [1, 2]. The particular landscape is unique to China, and some similar erosion landforms [3–5] are also found in other countries. Compared to other similar landform abroad, Benggang in China is mainly developed in hilly lands with large slopes, which chemical elements, erosion and development characteristics [6–8] are significantly different. Benggang can cause the massive loss of topsoil and the reduction in soil fertility, which can further lead to ecological ulcers in severe cases [9–11]. Approximately 239,125 gullies occurred in seven provincial-level administrative regions [12–14], such as Guangdong, Jiangxi, Fujian, Hunan, Hubei, Anhui and Guangxi Provinces. Therefore, theoretical guidance and data support are urgently needed to relieve this soil erosion and remediate the eco-environment.

**Fig 1:**
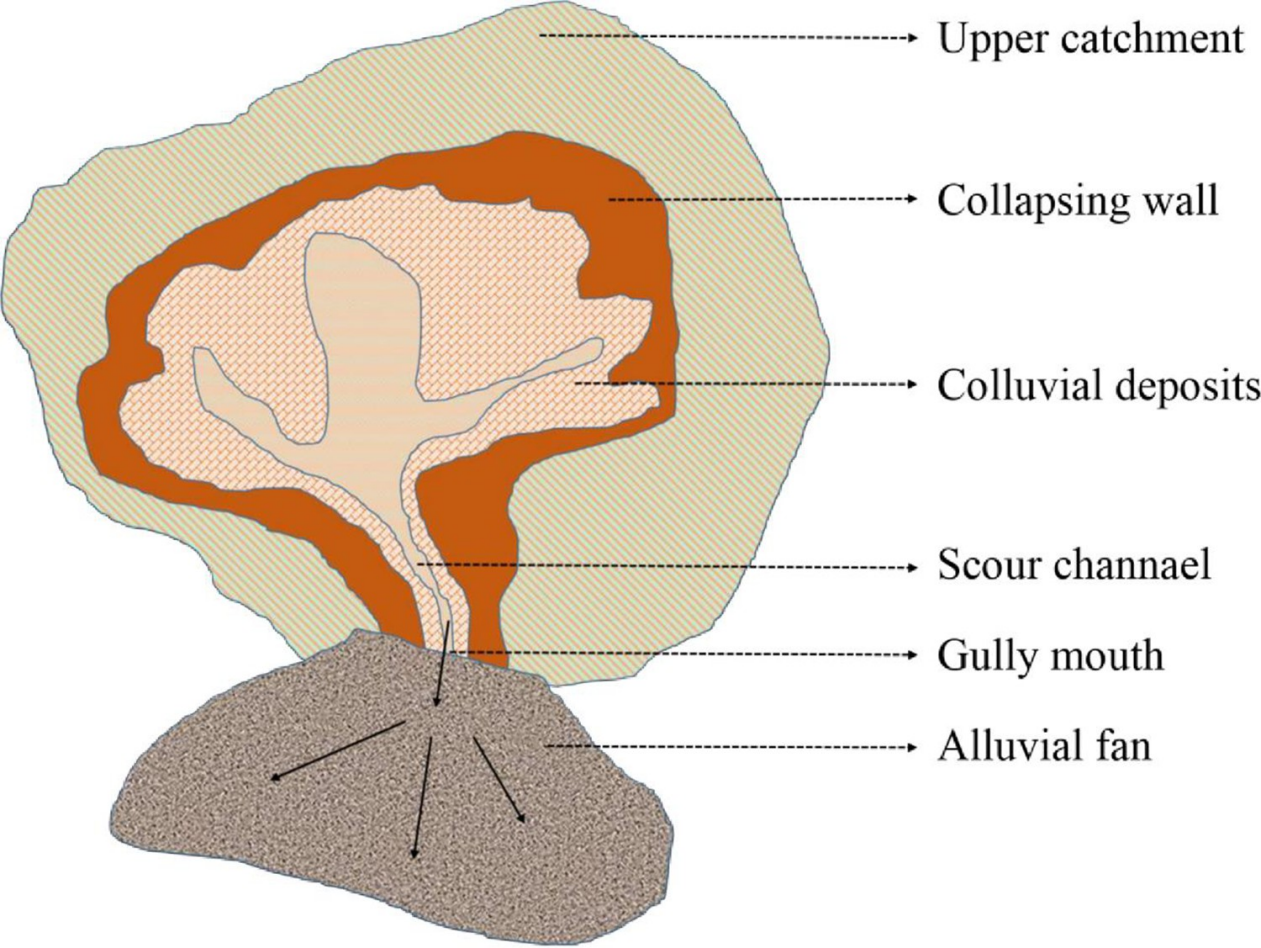
Schematic of a typical Benggang.

Generally, Benggang contains six parts [15–18], *i*.*e*., an upper catchment, collapsing walls, colluvial deposits, scour channels, a gully mouth and an alluvial fan (Fig 1). Previous works [19–22] have indicated that the instability of collapsing wall is a key step resulting in the occurrence and development of Benggang. Much attention has been paid to the factors affecting the erosion of collapsing wall, such as soil water content [23] and soil cementing materials [24]. Soil moisture, especially for the initial water content, is an important index to measure the soil mechanical properties of collapsing wall [13]. During rainfall, rainwater enters the collapsing wall through infiltration, which causes soil swelling due to the rapid increase in the water content. After rainfall, the soil moisture evaporates gradually and causes soil shrinkage with declining water content. Because of the frequent dry-wet cycles, the cracks caused by soil swelling and shrinkage gradually increase. Under the combined action of soil hydraulic stress and gravity, the surface of the collapsing wall is constantly flaking and collapsing, which eventually leads to the accelerated development of soil erosion. The change in soil water content can influence the concentration of ions, soil swelling and shrinkage, and further affect the interactions between soil particles. Nevertheless, the questions of how ionic concentration affect the soil swelling of collapsing wall are not clear still to now.

The soil colloidal particles are the most chemically active part of the soil [25]. Surface of soil colloidal particles has a large amount of electric charge and it can adsorb anions or cations to bind with the surrounding positive or negative charges. The ions on soil colloidal surface can migrate into soil solution through thermal motion, repulsive and attractive electric forces etc., thus forming a structure of electric double layer at the interface of the colloidal particles [26, 27]. Clay particles are negatively charged on the surface due to isomorphous substitution inside of clay structures and it can adsorb a large number of cations dispersed in mud to achieve hydration thus forming a hydration film on the surface of clay particles [26]. In the case of Kaolinitic clays and some other minerals in the clay fraction especially in tropical or subtropical regions with low pH clays may have positive charges rather than negative [28]. Hydration affects the stability of soil slope through the change of pore hydration state such as solutes and concentrations [28, 29], thereby causing the occurrence of natural geological hazards. When the proportion of clay in soil exceeds 10%, it will greatly influence soil mechanical properties [30]. For montmorillonite, hydration not only changes the thickness of electric double layer for soil colloidal particles but also reduces the electrostatic repulsion between adjacent particles due to the overlap of two electric layers. For kaolin, the water chemical state changes the physicochemical attraction among particles leading to the change in soil structure and further affects its physical and mechanical properties [31–34]. However, the surface properties of soil colloidal particles of collapsing wall and their relationship with the soil swelling behavior are still unclear.

Therefore, the objectives of this study were to (1) evaluate the effect of initial water content and sodium concentration on soil swelling rate of collapsing wall, (2) quantitatively calculate the thickness of shear plane (*x*_*s*_) of soil colloidal particles, an important physical and chemical parameter was obtained and (3) further study the correlation between surface properties of soil colloidal particle and soil swelling rates (*δ*_*s*_).

## Materials and methods

### Study area and sampling site

Longmen Town of Anxi County has become one of the most serious soil erosion areas in Fujian Province, south-east China [35]. There were 12,828 gullies in Anxi County, covering 49.28% of total gully area of Fujian Province. Longmen Town has 1,228 gullies, which accounts for 9.57% of total number of gullies in Anxi County. The sampling site (118°03′E, 24°57′N) is located on Yangkeng village of Longmen Town (Anxi County, Fujian Province) in the study area, which is a small valley basin and subtropical monsoon climate. Mean annual temperature and precipitation are 19°C and 1,800 mm, respectively. Rainfall is mainly concentrated from May to September, which is obviously affected by typhoon during summer. The soil is developed from acidic granite [36]. The mineral components mainly consisting of feldspar, quartz, mica and kaolinite minerals [16, 37].

### Soil samplings and analyses

**Fig 2:**
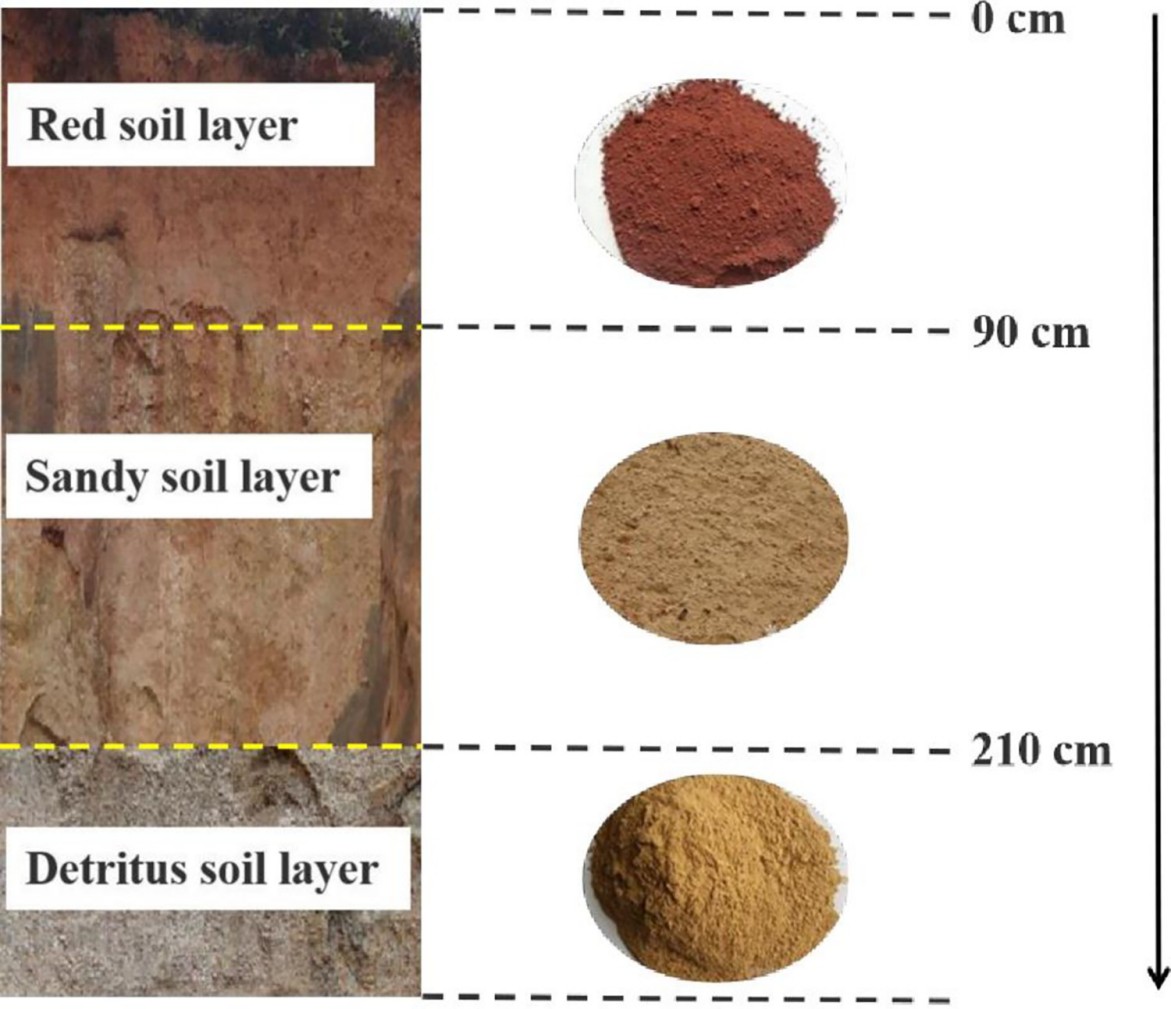
Distribution of different soil layers in collapsing wall.

Tested soil samples were collected along natural profile of a collapsing wall from a typical Benggang (Fig 2). According to sampling depth, collected soil was divided into red soil layer (0–90 cm), sandy soil layer (90–210 cm) and detritus soil layer (>210 cm) (Table 1) in this study.

**Table 1:** Basic information of sampling soil layers.

Soil layer	Depth	Soil description
	(cm)	
**Red soil**	0–90	Soil color is red, the texture is heavy, soil layer is compact and soil weathering is relatively complete, mostly feldspar and mica weathering, and coarse grain quartz content is less, there are some roots of *Dicranopteris dichotoma* and other plants distributed among them.
**Sandy soil**	90–210	The soil color is mainly light red and gray-white, with some black spots interspersed, soil is loose and easy to loose and contains more large-grain quartz.
**Detritus soil**	>210	Soil color is grayish white and loose, contains a lot of quartz sand, the incompletely weathered rock and quartz are closely combined, basically maintaining the original structure of granite.

All collected soil samples were air-dried naturally and passed through a 2 mm-sieve. Soil pH value (Soil-water ratio: 1:2.5) was measured by a STARTER 2100 pH meter (OHAUS Instruments Co., Ltd., Shanghai, China). A liquid-plastic combined tester (Shanghai Luda Experimental Instrumental Company, Shanghai, China) was used to test the liquid-plastic limit of soil. Soil bulk density (BD) was determined using weight method [38]. Cation-exchange capacity (CEC) was determined by ammonium acetate method [39]. Soil organic matter (SOM) was examined using an elemental analyzer (VarioMaxcube, Elementar Analysensysteme GmbH, Germany). Soil particle size distribution was measured by a BT-9300ST laser particle size distribution instrument (Dandong Bettersize Instruments Co., Ltd., Liaoning Province, China).

The tested soil clay samples were saturated with MgCl_2_ and KCl solutions and were mounted via smear-on glass slide technique for X-ray diffraction (XRD) analysis. Mg-saturated clays were examined at 25°C before and after glycerol solvation. K-saturated clays were examined at 25°C and after heating at 110, 250, 350, 450 and 550°C for 2 hours. Oriented clay mineral aggregates were examined using an X-ray diffractometer (Ultima IV, Rigaku Corporation, Japan) with CuKα radiation (λ = 1.5418 Å) generated at 40 kV and 40 mA [40]. The XRD patterns were examined over the range of 3–40°2Ɵ with a scan speed of 1° min^−1^ [41]. Semi-quantitative analysis of clay was performed according to the methods [42] reported by Pai et al. All tests were conducted in triplicate.

### Measurement of soil swelling rates

Soil bulk density of remolded soil was set at 1.40 g cm^-3^. Effects of initial water content and electrolyte concentration on soil swelling characteristics of collapsing wall were evaluated. Initial water content was ranged from 15% to 35% for red soil layer and from 15% to 30% for sandy and detritus soil layers. Interval of initial water content was set at 5%. Previous studies have shown that sodium ions can enhance soil water holding capacity. The increase of water-holding capacity will inevitably have an impact on soil expansion. The effects of NaNO_3_ concentration (1×10^−4^, 1×10^−3^, 1×10^−2^, 1×10^−1^ and 1×10° mol L^-1^) on soil swelling rate under extremely low water content (5%) were studied. The data was collected and recorded continuously for 24 hours.

**Fig 3:**
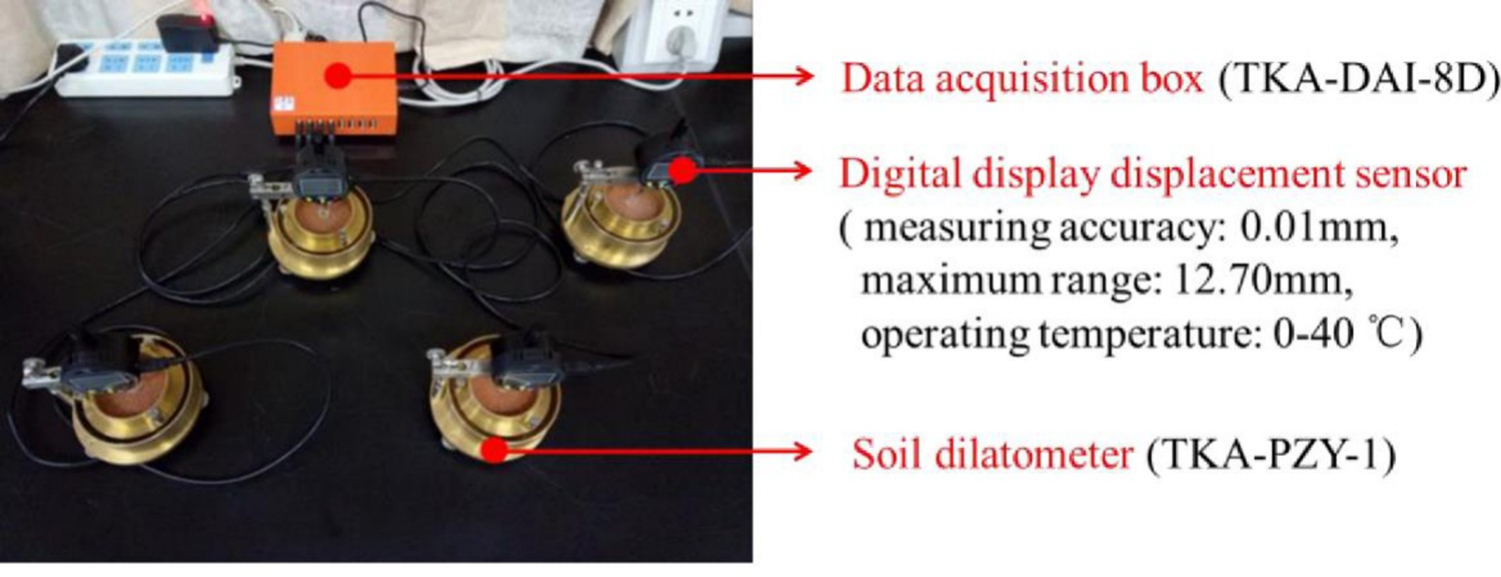
Measurement device of one-dimensional linear swelling.

Soil swelling tests were carried out according to the Specification of Soil Test [43]. First, thin petroleum jelly was evenly applied to the inner wall of the stainless-steel ring, and remolded soil was prepared with a compaction hammer and packed until the desired soil bulk density. The porous stone was then placed in a soil dilatometer (TKA-PZY-1, Nanjing TKA Technology Co., Ltd., Shanghai, China) (Fig 3), and stainless-steel ring with soil sample was installed on the base by a pressure ring. Volume of soil container was 60 cm^3^, and the container was 20 mm height. After 24 hours of continuously monitoring, the data was collected and the remaining solution in container was extracted. Soil sample was oven-dried at 105°C for 8 hours and weighed. Each treatment was performed in triplicate.

Soil swelling rate (*δ*_*s*_) was used to characterize the magnitude of soil swelling [44] and was calculated by following equation:

$$\delta_{s}=\frac{h_{t}-h_{0}}{h_{i}}\times 100\%$$ (1)

where *δ*_*s*_ was soil swelling rate (%); *h*_0_ and *h*_*t*_ were display values (mm) of the displacement sensor at beginning time 0 and the ending time *t*; *h*_*i*_ was initial height (mm) of the sample.

### Collection of soil colloidal particles

Fifty grams of air-dried soil sample was weighed and placed in a glass beaker. Soil organic matter was removed by 30% H_2_O_2_ and it was heated at 70°C on an electric heating plate [38]. Then, soil sample without organic matter was dispersed using a 5% sodium hexa-metaphosphate solution and stirred by an electric mixer (AM250W-T, Shanghai ANGNI Instruments Co., Ltd., Shanghai, China) at 500 r min^-1^ for 10 min. The dispersed suspension was transferred into a measuring cylinder. After settling for 20 hours, soil colloidal particle suspension was collected according to Stokes’ law [40]. Soil colloidal particles were obtained by centrifugation and then freeze-dried.

### Sodium/Calcium exchange equilibrium to determine soil colloidal surface properties of charged particles

Combined determination method [45] was used to measure soil colloidal surface properties of charged particles. The operation steps were as follows: First, soil colloidal particles were saturated by HCl solution (0.1 mol L^-1^). Then, two grams of hydrogen-saturated sample was weighed and placed into centrifuge bottle, and 15 mL of Ca(OH)_2_ (0.02 mol L^-1^) was added and oscillated for 12 hours. Finally, 15 mL of NaOH (0.02 mol L^-1^) was added and oscillated for 24 hours, and its pH was measured after oscillation. The pH of the suspension was adjusted with HCl (1 mol L^-1^). After 12 hours of oscillation, the adjusted pH of suspension was measured and the pH value was stabilized at neutral. The supernatant was collected by centrifugation, and Ca^2+^ and Na^+^ concentrations in supernatant were determined by an atomic absorption spectrometer (PinAAcle 900, PerkinElmer Enterprise Management Co., Ltd., USA) and a flame photometer (FP6400A, Hebei Bohui Instrument Co., Ltd., Hebei Province, China), respectively. The adsorbing amount of Na^+^ (*N*_*Na*_) or Ca^2+^ (*N*_*Ca*_) on the soil colloidal particle surface was obtained by subtracting the measured concentration value from the concentration of the bulk solution according to previous report [45]. Adsorbing capacity of Na^+^ and Ca^2+^ was used to calculate the parameters of clay surface charge properties.

### Zeta potential tests for soil colloidal particles

Zeta potential (*ζ*) was measured with a zeta potentiometer (NanoBrook Omni, Brookhaven Instruments Corporation, NY). The procedure was performed as follows. Ten milligrams of soil colloidal particle were weighed and put into a centrifuge tube, and then 10 mL of NaNO_3_ solution at different concentrations was added as electrolyte (suspension concentration ≤1 mg mL^-1^). The electrolyte concentrations of NaNO_3_ were set as 1×10^−4^, 1×10^−3^, 1×10^−2^, 1×10^−1^ and 1×10° mol L^-1^. Each test was performed in triplicate.

### Calculation of surface property parameters of soil colloidal particles

Surface potential (*φ*_0_) and zeta potential (*ζ*) of soil colloidal particles were plug into the following equations to obtain thickness (*x*_*s*_) of shear plane of soil colloidal particles. The thickness of shear plane of soil colloidal particles is an important surface property parameter. The surface charge properties including *φ*_0_, *S*, *SCN*, *σ*_0_ and *x*_*s*_ of colloidal particles for different soils were calculated using the combined determination method [45] as follows:

$$\varphi_{0}=\frac{2RT}{(2\beta_{Ca}-\beta_{Na})F}\ln\frac{c_{Ca}^{0}N_{Na}}{c_{Na}^{0}N_{Ca}}$$ (2)

where *φ*_0_ was surface potential (mV) in a mixture solution of Na^+^ and Ca^2+^; *R* was gas constant (J K^-1^ mol^-1^); *T* was absolute temperature (K); *F* was Faraday constant (C mol^-1^); $c_{Ca}^{0}$ and $c_{Na}^{0}$ were concentrations of Ca^2+^ and Na^+^ in bulk solution (mol L^-1^), respectively; *N*_*Ca*_ and *N*_*Na*_ were quantities of Ca^2+^ and Na^+^ adsorbed on particle surface; and *β*_*Ca*_ = 0.0213ln*I*^1/2^+1.2331 (where *I* was ionic strength in moles per liter) and *β*_*Na*_ = 2−*β*_*Ca*_ were modification factors.

$$S=\frac{N_{Na}\kappa }{mc_{Ca}^{0}}e\frac{\beta_{Na}F\varphi_{0}}{2RT}$$ (3)

where *S* was specific surface area (m^2^ g^-1^) of colloidal particles; *κ* was Debye-Hückel parameter (dm^-1^); 1/*κ* was thickness of electric double layer; $m=0.5259\ln(c_{Na}^{0}/c_{Ca}^{0})+1.992$ was modification factor. Additionally, *κ* was defined as follows:

$$\kappa =\sqrt{\frac{8\pi F^{2}Z^{2}c}{\varepsilon RT}}$$ (4)

where *Z* was valence number of counterion; *c* and *ε*were equilibrium concentration (mol L^-1^) of cations in bulk solution and dielectric constant of water.

$$SCN=N_{Na}+2N_{Ca}$$ (5)

where *SCN* was surface charge number (mol kg^-1^) of colloidal particles.

$$\sigma_{0}=\frac{SCN\times F\times 10^{-5}}{S}$$ (6)

where *σ*_0_ was surface charge density (C m^-2^).

$$\varphi_{0}=\frac{2RT}{F}\ln(\frac{2c}{\sigma_{0}\kappa })$$ (7)

where *φ*_0_ was surface potential (mV) of colloidal particles.

$$x_{s}=-\frac{1}{\kappa }\ln[\frac{1-e^{\xi F/2RT}}{(1+e^{\xi F/2RT})\lambda }]$$ (8)

where *x*_*s*_ was thickness (nm) of shear plane for colloidal particles and *ζ* was zeta potential (mV) of colloidal particles, *λ* was defined as follows:

$$\lambda =\frac{1-e^{F\varphi_{0}/2RT}}{1+e^{F\varphi_{0}/2RT}}$$ (9)

where *λ* was a parameter calculated from surface potential of colloidal particles.

### Statistical analysis

Pearson correlation analysis was used to determine the correlation coefficient between soil swelling rate of different soil layers and shear plane thickness of colloidal particles. All tests were performed using the statistical program SPSS 18.0.

## Results

### Soil physicochemical properties

General physicochemical properties of the soil samples were presented in Table 2. The pH values of red, sandy and detritus soil layers were 4.13, 4.99 and 5.23, respectively. It was found that soil organic matter contents decreased with increasing soil depths. Detritus soil layer had the largest bulk density with a value of 1.45 g cm^-3^, which was higher than that of the red (1.33 g cm^-3^) and sandy (1.31 g cm^-3^) soil layers. The FeO content of red, sandy and detritus soil layers were 0.20, 0.06 and 0.04 g kg^-1^, respectively. Red soil layer displayed the highest CEC value with 30.12 cmol kg^-1^, which was 7 and 11 times higher than those of sandy and detritus soil layers, respectively. Plastic limits of red, sandy and detritus soil layers were 35.27, 29.89 and 30.17, and liquid limits were 75.05, 45.52 and 44.01, respectively. Clay and silt contents decreased with increasing soil depths, and it was opposite in sand contents. Moreover, the XRD results revealed that red soil layer consists of four kinds of minerals: kaolinite, illite, gibbsite and hydroxy-interlayered vermiculite (HIV) (Table 3). HIV was not detected in sandy soil layer. Detritus soil layer only contains kaolinite and illite. Notably, kaolinite dominated the relative over 80% of minerals for all soil layers of collapsing wall.

**Table 2:** General physical and chemical properties of tested soils.

Soil layer	pH	SOM ^a^	FeO	CEC ^b^	BD ^c^	Plastic limit	Liquid limit	Clay	Silt	Sand
	(Soil:H_2_O = 1:2.5)	(g kg^-1^)	(g kg^-1^)	(cmol kg^-1^)	(g cm^-3^)			(%)	(%)	(%)
**Red soil**	4.13±0.28	7.41±1.50	0.20±0.0039	30.12±2.22	1.33±0.03	35.27±1.72	75.05±3.43	7.59	42.01	50.40
**Sandy soil**	4.99±0.16	4.78±0.08	0.06±0.0070	3.85±0.65	1.31±0.01	29.89±0.63	45.52±2.81	2.89	28.34	68.77
**Detritus soil**	5.23±0.19	4.59±0.58	0.04±0.0097	2.69±0.11	1.45±0.06	30.17±2.03	44.01±1.35	2.01	24.11	73.88

^a^ Soil organic matter.

^b^ Cation exchange capacity.

^c^ Bulk density.

**Table 3:** Semi-quantitative analysis of clay mineral compositions in the tested soils (<2 um).

Soil layer	Kaolinite	Illite	Gibbsite	HIV ^a^
	(%)	(%)	(%)	(%)
**Red soil**	80.65	5.22	6.09	8.04
**Sandy soil**	91.46	6.74	1.80	—^b^
**Detritus soil**	88.68	11.32	—	—

^a^ Hydroxy-interlayered vermiculite.

^b^ Indicates not detected.

### Effects of initial water contents and NaNO_3_ concentrations on the soil swelling rates

**Fig 4:**
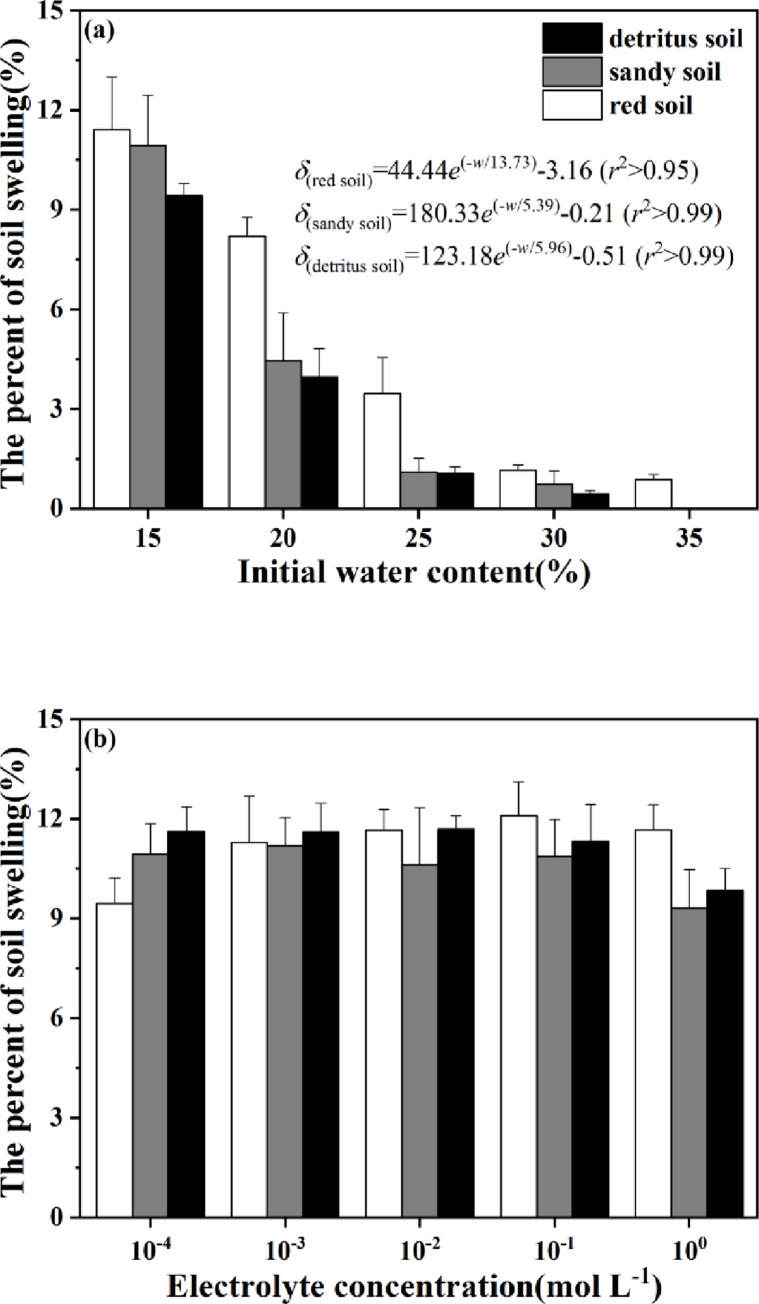
Effects of different (a) initial water contents and (b) electrolyte concentrations on soil swelling rate for tested soils.

Soil swelling rate (*δ*_*s*_) reflected the degree of soil swelling and deformation, which was closely related to hydrophilic ability of soil particles. As shown in Fig 4A, the *δ*_*s*_ values decreased with increasing initial water contents (*w*) for red, sandy and detritus soil layers. When the water content was 15%, the swelling rate of red soil layer was 11.41%, which was the largest compared with sandy (10.93%) and detritus (9.42%) soil layers. Relationship between initial water content and soil swelling rate was a function with an exponential decline, *i*.*e*., $\delta_{red}=44.44e^{\left(-w/13.73\right)}-3.16$ (*r*^2^>0.95) for red soil layer, $\delta_{sandy}=180.33e^{\left(-w/5.39\right)}-0.21$ (*r*^2^>0.99) for sandy soil layer, and $\delta_{\det ritus}=123.18e^{\left(-w/5.96\right)}-0.51$ (*r*^2^>0.99) for detritus soil layer.

Generally, soil initial water contents affected the ionic concentrations in the collapsing wall of Benggang. Fig 4B showed effects of NaNO_3_ concentrations on *δ*_*s*_ values. When NaNO_3_ concentration ranged from 10^−4^ to 10^−3^ mol L^-1^, the *δ*_*s*_ values in detritus soil layer were higher than those in the sandy and red soil layer. Interestingly, similar *δ*_*s*_ values were found in red and detritus soil layers when exposed to 10^−2^ mol L^-1^ NaNO_3_ solution. Once the NaNO_3_ concentration was over 10^−1^ mol L^-1^, red soil layer displayed the highest swelling rate among three soil layers.

### Effects of the NaNO_3_ concentration on the zeta and surface potentials

**Fig 5:**
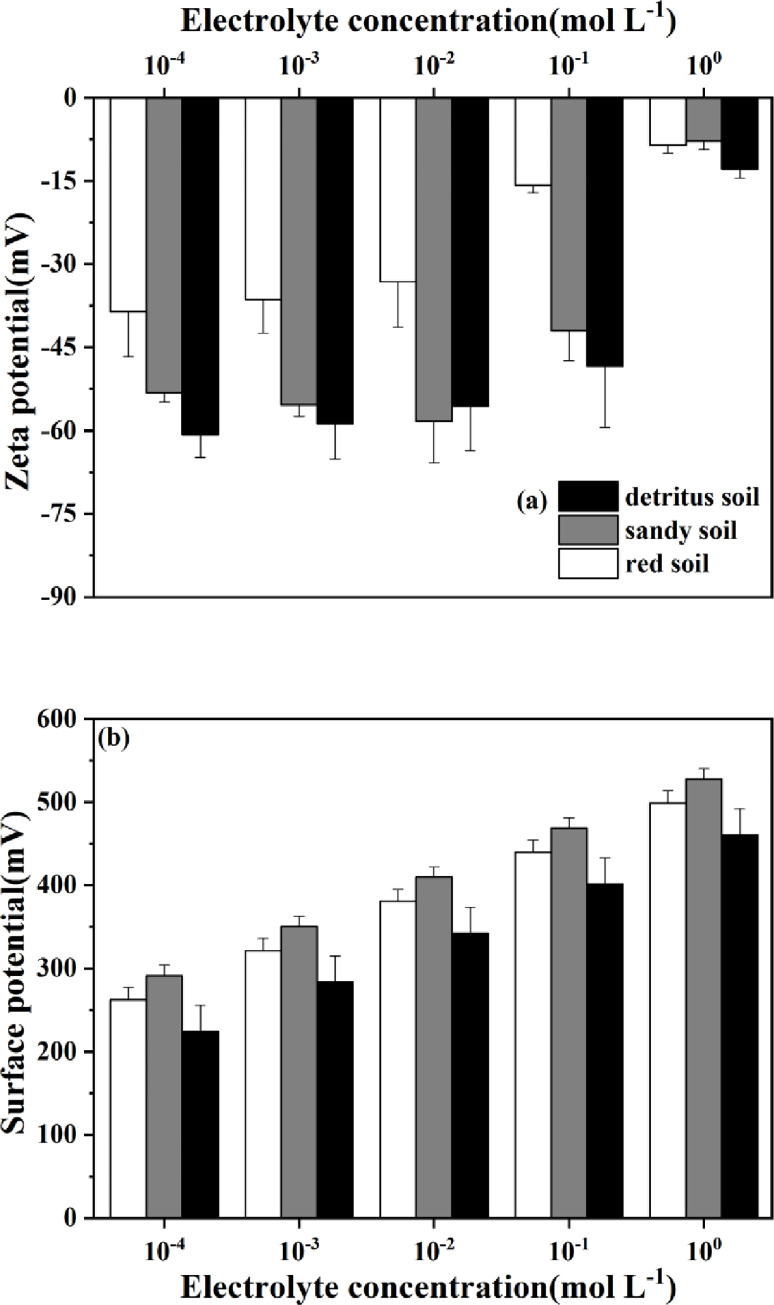
Relationship between (a) zeta potential and (b) surface potential with electrolyte concentrations for tested soils.

Surface potentials (*φ*_0_) and corresponding thickness of shear plane (*x*_*s*_) for soil colloidal particles of different soil layers in collapsing wall were investigated. Surface electrochemical characteristics of soil colloid particles reflected the soil hydrophilicity. As indicated in Fig 5A, zeta potential (*ζ*) of red and detritus soil colloid particles increased with increasing NaNO_3_ concentrations. Moreover, zeta potential of colloid particles in sandy soil layer first decreased and then increased. And *ζ* value of red soil layer was mostly higher than that of sandy and detritus soil layers.

Surface charge density (*σ*_0_) was calculated by Eq 2, and the parameters of soil surface electrochemical properties, including ionic strength (*I*), specific surface area (*S*) and surface charge density, were all shown in Table 4. Based on these indicators and parameters, the surface potential (*φ*_0_) of soil colloidal particles in a 1:1 electrolyte system was obtained by Eq 3. Surface potential of soil colloidal particles in different soil layers increased with increasing NaNO_3_ concentrations (Fig 5B). The *φ*_0_ values in sandy soil layer were higher than those in red and detritus soil layers in any concentrations of NaNO_3_ solution. Significant differences were observed between *ζ* and *φ*_0_ at the same electrolyte concentration. Thickness of shear plane was far from Stern plane on surface of soil colloidal particles. However, *φ*_0_ value of soil colloidal particles collected from the collapsing wall ranged from 224.15±31.37 to 527.81±12.51 mV.

**Table 4:** Soil surface properties of colloidal particles in different soil layers under neutral pH conditions.

Soil layer	$c_{Ca}^{0}$ ^a^	$c_{Na}^{0}$ ^b^	*N*_*Ca*_^c^	*N*_*Na*_^d^	1/*κ* ^e^	*I* ^f^	*φ*_0_^g^	*S* ^h^	*σ*_0_^i^
	(10^−3^ mol L^-1^)		(10^−3^ mol kg^-1^)		(nm)	(10^−2^ mol L^-1^)	(mV)	(10^2^ m^2^ kg^-1^)	(10^−2^ C m^-2^)
**Red soil**	3.89±0.79	9.12±0.07	5.99±0.79	0.76±0.07	3.085±0.001	2.99±0.04	-82.79±8.94	34.74±6.47	3.69±1.07
**Sandy soil**	6.21±0.11	9.24±0.07	3.60±0.11	0.57±0.07	3.097±0.003	3.34±0.10	-63.45±5.39	37.74±7.96	2.08±0.54
**Detritus soil**	6.28±0.69	9.54±0.07	3.48±0.70	0.22±0.07	3.105±0.002	3.58±0.08	-90.55±13.73	10.11±4.91	8.69±4.78

^a^ and ^b^ are concentrations of Ca^2+^ and Na^+^ in bulk solution, respectively.

^c^ and ^d^ are quantities of Ca^2+^ and Na^+^ adsorbed on the particle surface, respectively.

^e^ Thickness of electric double layer.

^f^ Ionic strength.

^g^ Surface potential in a mixture solution of Na^+^ and Ca^2+^.

^h^ Specific surface area of colloidal particles.

^i^ Surface charge density.

### Effects of the NaNO_3_ concentration on the thickness of electric double layer and shear plane

**Fig 6:**
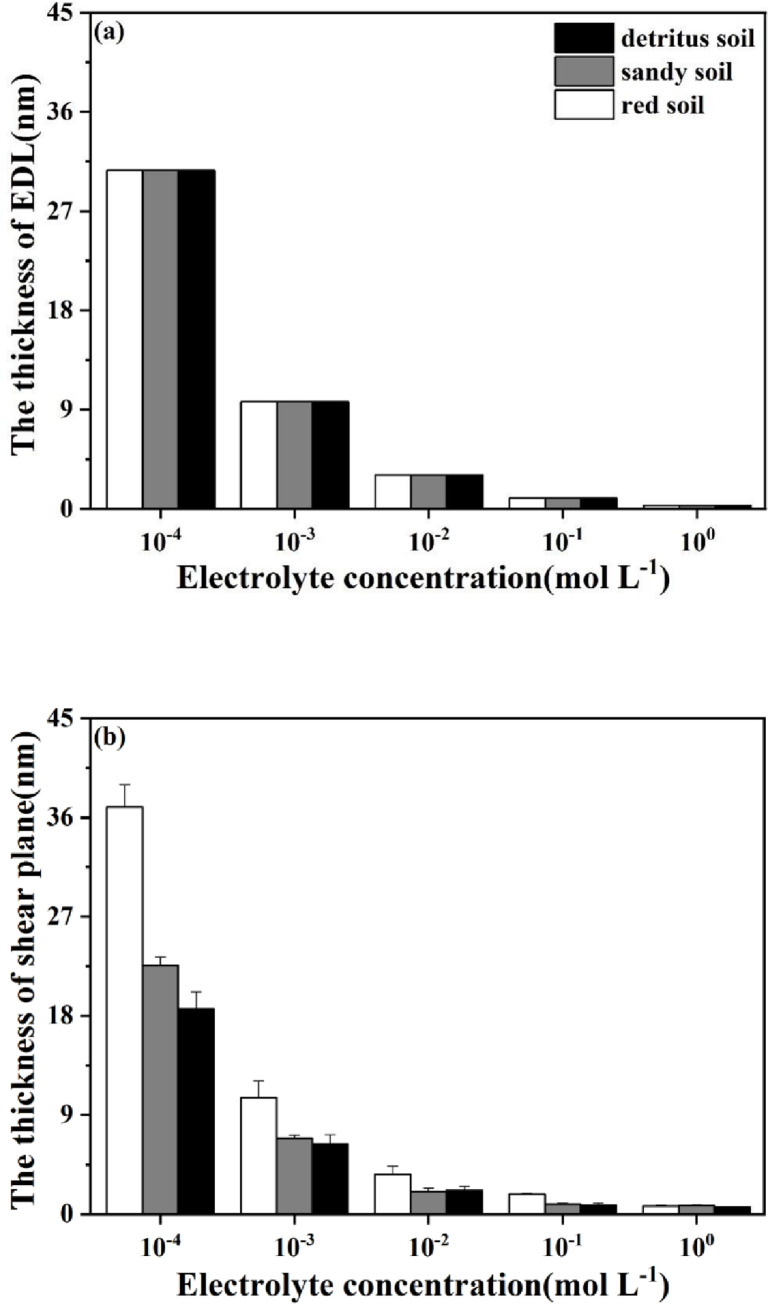
Thickness of (a) electrical double layer and (b) shear plane under different electrolyte concentrations for tested soils.

According to the electric double layer theory [26], the thickness of shear plane (*x*_*s*_) and electric double layer (1/*κ*) in a 1:1 electrolyte system were calculated by Eqs 4 and 5. When NaNO_3_ concentration increased from 10^−4^ to 10° mol L^-1^, the 1/*κ* value in all soil layers decreased from 30.70 to 0.31 nm (Fig 6A). Furthermore, *x*_*s*_ value was reduced from 39.69 to 0.76 nm in red soil layer, from 22.56 to 0.79 nm in sandy soil layer, and from 18.61 to 0.64 nm in detritus soil layer (Fig 6B). For thickness of shear plane, significant differences were observed among each soil layer while NaNO_3_ concentration was extremely low (10^−4^ mol L^-1^). Nevertheless, no difference in *x*_*s*_ value was observed among each soil layer once the NaNO_3_ concentration was 1×10° mol L^-1^. The difference in *x*_*s*_ value among different soil layers was reduced with increasing NaNO_3_ concentration.

### Pearson correlation between the soil swelling rates and soil colloidal particle surface properties

Pearson correlation between the soil swelling rates and soil colloidal surface properties were shown in Table 5. The *δ*_*s*_ of red soil layer exhibited no correlation with *φ*_0_, and no significantly negative correlations between *δ*_*s*_ and *φ*_0_ in sandy and detritus soil layers were observed. The *δ*_*s*_ of red soil layer exhibited an extremely significant negative correlation with the 1/*κ* (*P*<0.01), and no correlation between *δ*_*s*_ and 1/*κ* with sandy and detritus soil layers was found. The *δ*_*s*_ of sandy soil layer demonstrated a significant negative correlation with *ζ* (*P*<0.05), and the *δ*_*s*_ of detritus soil layer showed an extremely significant negative correlation with *ζ* (*P*<0.01). However, no correlation between *δ*_*s*_ and *ζ* was found in red soil layer. In particular, the relationship between *δ*_*s*_ and *x*_*s*_ showed an extremely significant negative correlation for red soil layer and no correlation for sandy and detritus soil layers. The absolute values of correlation coefficient of red soil layer were much greater than that those of sandy and detritus soil layers.

**Table 5:** Pearson correlation coefficients between partial surface electrochemical parameters of colloidal particles for different soils and soil swelling rates (*n* = 15).

Soil layer	Parameter	*φ*_0_^a^	*ζ* ^b^	1/*κ* ^c^	*x*_*s*_^d^
**red soil**	*δ*_*s*_^e^	0.80	0.64	-0.99** ^f^	-0.99**
**sandy soil**		-0.76	-0.91* ^g^	0.44	0.42
**detritus soil**		-0.78	-0.99**	0.44	0.43

^a^ Surface potential of colloidal particles (mV).

^b^ Zeta potential (mV).

^c^ Thickness of the electric double layer (nm).

^d^ Thickness of the shear plane (nm).

^e^ Soil linear swelling rate (%).

^f^ Indicates significant values at the 0.01 level (two-tailed).

^g^ Indicates significant values at the 0.05 level (two-tailed).

## Discussion

Previous study [46] reported that initial water contents had a significant effect on soil swelling rates. The initial water contents affected the electrolyte concentrations in soil thus leading to non-ignorable changes in the thickness of shear plane of electric double layer [45]. Therefore, we examined the effects of different initial water contents and NaNO_3_ concentrations on *δ*_*s*_ value for all soil layers including red, sandy and detritus soil layers. It was found that the tested samples of collapsing wall exhibited distinct soil swelling behaviors under the same initial water content (Fig 4A). The reason was that physical and chemical indexes directly related to the soil hydrophilicity, such as liquid limit, CEC value and clay content, decreased with increasing soil depth.

10^−2^ mol L^-1^ was a critical concentration point [47, 48] where both the DLVO force and net force acted as functions of electrolyte concentration. When the concentration of NaNO_3_ varied from 10^−4^ to 10° mol L^-1^, the amplitude of variation in the swelling rates did not exceed 3% for all soil layers. The *δ*_*s*_ value of red soil layer increased with elevated NaNO_3_ concentration, and the opposite results were observed in sandy and detritus soil layers. Occurrence of these interesting phenomena might be related to the distinct soil nature of different soil layers in collapsing wall. Another important reason was that electrolyte concentration changed internal forces among soil particles and further affected the thickness of shear plane in electric double layer for soil colloid particles [49].

For the *ζ* values of all soil samples collected from the collapsing wall, they ranged from -7.84±1.53 to -60.70±4.12 mV (Fig 5A), which were in accordance with the data of -15 ~ -60 mV previously reported [50, 51]. However, no significant difference in *ζ* values of colloid particle was found between sandy and detritus soil layers, indicating that the stability of colloidal dispersion had no obvious change in these two soil layers. It was noting that the *φ*_0_ values of soil colloidal particles collected from the collapsing wall was positive, which was completely different from the negative potentials obtained from purple soil [52, 53]. This was mostly due to differences in soil mineral composition. That was, the main mineral composition of purple soil was calcium carbonate, which did not show the characteristics of desilication and aluminization. In contrast, the granite residual laterite in South China contained Fe-oxides (0.04 ~ 0.20 g kg^-1^) (Table 2) coated kaolinite (more than 75%) which displayed strong desilication and aluminization [18].

At low electrolyte concentrations from 10^−4^ to 10^−3^ mol L^-1^, the variation of *φ*_0_ value was much greater than that of *ζ* value and the differences in the values of *φ*_0_ and *ζ* decreased with the increasing soil depths (Fig 5). According to Eqs 8 and 9, the value of thickness of shear plane (*x*_*s*_) was determined as functions of *φ*_0_, *ζ* and 1/*κ*. Moreover, the values of 1/*κ* decreased with the elevated electrolyte concentrations. In this case, the estimated values of *x*_*s*_ obtained from different electrolyte concentrations obviously decreased with increasing soil depths. The difference was non-significant in their values but was much greater than those of *φ*_0_ and *ζ*, which were consistent with the variation tendency of *δ*_*s*_ mentioned above. On the other side, the discrepancy in the values of *x*_*s*_ and 1/*κ* gradually reduced with increasing NaNO_3_ concentrations from red, sandy to detritus soil layers. This result indicated that the compression of 1/*κ* value for colloidal particles was conducive to the particle proximity, the increment in Van der Waals force and condensation among soil particles [54, 55].

In a solution of sodium nitrate, the condensation of soil colloidal particles reflected the inhibition of soil swelling at macroscopic level. Thus, combined with the observed results above (Figs 4B and 6B), it could be inferred that the condensation of soil colloidal particles had an inhibitory effect on soil swelling rates and a certain correlation existed between the thickness of shear plane (*x*_*s*_) and soil swelling rate (*δ*_*s*_). In fact, the relationship between the values of *x*_*s*_ and *δ*_*s*_ was significantly different from all soil layers (Table 5). This result might be related to the decreasing distribution of Fe-oxide content in the collapsing wall with soil depths [18]. Some studies [56–58] had suggested that Fe-oxide played an important role, which coated on the surface of kaolinite grains in maintaining soil structural stability. Loose subsoil (*i*.*e*., sandy or detritus soil layer) with low Fe-oxide contents usually disintegrated first after rainfall, and then the structurally stable red soil with high Fe-oxide contents collapsed, which finally caused soil erosion.

## Conclusion

In this study, upper red soil layer of collapsing wall had superior physical and chemical properties, and the kaolinite was the dominant mineral. Relationship between initial water contents and soil swelling rates was a function with exponential decline. Under different NaNO_3_ concentrations, the maximum variation in soil swelling rates of all soil layers was not more than 3%. Relationship between soil swelling rates and thickness of shear plane of fine clay fractions showed an extremely significant negative correlation for red soil layer and no correlation for sandy and detritus soil layers. It was related with decreasing distribution of Fe-oxide content with the soil depths. These findings provided a new perspective on the relationship between surface properties of soil colloidal particles affected by Na^+^ concentrations and soil swelling behaviors, and indicated that the soil particle interaction played a crucial role in the development and occurrence of Benggang. It will be helpful in understanding and interpreting the mechanisms of Benggang. Future work will focus on the effects of internal forces between soil particles driven by electrolyte concentrations and types on swelling and shrinkage behaviors of different soil layers in collapsing wall of Benggang. Relationship between soil internal forces, swelling and shrinkage behaviors of collapsing wall also deserves further in-depth study.
